# Expression of Glioma-associated oncogene homolog 1 as biomarker with sonidegib in advanced basal cell carcinoma

**DOI:** 10.18632/oncotarget.27735

**Published:** 2020-09-15

**Authors:** Reinhard Dummer, Li Liu, Nicholas Squittieri, Ralf Gutzmer, John Lear

**Affiliations:** ^1^Department of Dermatology, University of Zürich, Skin Cancer Center, University Hospital, Zürich, Switzerland; ^2^Sun Pharmaceutical Industries, Inc., Princeton, NJ, USA; ^3^Skin Cancer Center Hannover, Department of Dermatology, Hannover Medical School, Hannover, Germany; ^4^Manchester Academic Health Science Centre, Manchester University and Salford Royal NHS Trust, Manchester, UK

**Keywords:** biomarker, *Glioma-associated oncogene homolog 1*, basal cell carcinoma, Hedgehog pathway inhibitor, sonidegib

## Abstract

The pivotal BOLT (Basal cell carcinoma Outcomes with LDE225 [sonidegib] Treatment) study established the durable efficacy and manageable toxicity of sonidegib 200 mg once daily (QD) through 42 months in patients with advanced basal cell carcinoma (BCC). This secondary analysis used expression of *Glioma-associated oncogene homolog 1 (GLI1)* as a biomarker to assess the extent of Hedgehog pathway inhibition by sonidegib in patients with locally advanced BCC (laBCC) and metastatic BCC (mBCC). The study enrolled 230 patients, 79 and 151 receiving sonidegib 200 and 800 mg QD, respectively. At week 17, *GLI1* expression was reduced from baseline by a median percentage (95% confidence interval) of 88.7% (54.6%–93.0%) and 97.0% (77.5%–98.9%) for aggressive laBCC, 97.5% (80.3%–98.8%) and 95.0% (80.7%–97.5%) for nonaggressive laBCC, and 99.1% (96.4%–99.6%) and 99.3% (95.9%–99.9%) for mBCC in the 200 and 800 mg groups, respectively. Substantial repression of *GLI1* was observed in patient subgroups stratified by age, sex, BCC cytological subtype, Eastern Cooperative Oncology Group performance status, lesion site, baseline number of BCCs, and prior radiotherapy. Results support further studies on the inhibition of Hedgehog pathway genes by sonidegib in patients with laBCC and mBCC.

## INTRODUCTION

Basal cell carcinoma (BCC) is the most common skin malignancy, affecting more than 10 million new patients annually worldwide and with an incidence that increases by approximately 1% each year [1–3]. Most BCCs are treated with surgery, with a favorable prognosis [4]. In cases of locally advanced BCC (laBCC) where surgery is contraindicated, inhibition of the Hedgehog pathway is one of the few approved and recommended treatment options [4, 5].

Most BCCs exhibit constitutive activation of the Hedgehog pathway due to mutations in pathway members, most often *Patched 1 (PTCH1)* and *Smoothened (SMO)* [6–8]. Expression of the transcription factor *Glioma-associated oncogene homolog 1* (*GLI1*) is a biomarker for Hedgehog pathway activation [9].

Sonidegib, a Hedgehog pathway inhibitor (HHI), blocks pathway signaling by selective inhibition of *SMO* [10]. It is approved in the US, EU, Switzerland, and Australia for the treatment of adult patients with laBCC not amenable to curative surgery or radiotherapy [11–14]. In Switzerland and Australia, sonidegib is also approved for the treatment of metastatic BCC (mBCC) [13, 14].

The efficacy and safety of 2 doses of sonidegib (200 and 800 mg once daily [QD]) were assessed through 42 months of treatment in patients with advanced BCC in the Basal cell carcinoma Outcomes with LDE225 (sonidegib) Treatment (BOLT) study (NCT01327053) [15–18]. The primary analysis at 6 months showed objective response rate (ORR) (95% confidence interval [CI]) of 43% (28%–59%) and 38% (28%–48%) in laBCC and 15% (2%–45%) and 17% (5%–39%) in mBCC for the 200 and 800 mg QD doses, respectively [18]. The final analysis at 42 months was the longest clinical trial follow-up to date with an HHI and reported ORR (95% CI) of 56% (43%–68%) and 46% (37%–55%) in laBCC and 8% (< 1%–36%) and 17% (5%–39%) in mBCC for 200 and 800 mg QD, respectively [15]. At 42 months, adverse events (AEs) with the approved 200 mg QD dose were mostly Grade 1 or 2, manageable, and reversible with dose interruptions [15]. While some results regarding *GLI1* expression associations with clinical outcomes were reported in the BOLT primary analysis [18], here we report the complete secondary biomarker analysis of Hedgehog pathway inhibition with sonidegib from the BOLT study.

## RESULTS

### Patient disposition, demographics, and clinical characteristics

Overall, 230 patients were enrolled in the study, of whom 79 and 151 were randomized to sonidegib 200 and 800 mg QD, respectively (Supplementary Figure 1). At the time of data cutoff for biomarker analysis (6 months), 39 (49.4%) patients in the 200 mg group and 46 (30.5%) patients in the 800 mg group were still receiving treatment. The biomarker population included 67 and 83 patients randomized to sonidegib 200 and 800 mg QD, respectively. AEs were the most common reason for discontinuation, reported for 16 (20.3%) and 48 (31.8%) discontinuing patients in the 200 and 800 mg group, respectively.

Patient demographics and clinical characteristics were similar between the biomarker and intent-to-treat (ITT) populations (Supplementary Table 1). The biomarker population was 62.7% and 65.1% male with mean (standard deviation [SD]) age of 65.6 (15.6) and 64.0 (14.9) years for the 200 and 800 mg groups, respectively. The ITT population was 60.8% and 63.6% male with mean (SD) age of 65.6 (15.7) and 63.6 (14.6) years for the 200 and 800 mg groups, respectively. Patients with laBCC comprised 91.0% and 91.6% of the biomarker population, and 83.5% and 84.8% of the ITT population for the 200 and 800 mg groups, respectively.

### Reduction of GLI1 expression levels

Median (95% CI) *GLI1* expression at baseline was −2.64 (−3.22, −2.27) and −3.04 (−3.21, −2.56) for 200 and 800 mg groups, respectively. Longitudinal analyses showed substantial reductions in *GLI1* expression from baseline. Expression was reduced by a median (95% CI) of 87.4% (77.0%–96.1%) and 96.2% (94.1%–98.4%) at week 9, 92.7% (78.4%–96.8%) and 95.8% (91.8%–98.2%) at week 17, and 93.0% (33.1%–97.6%) and 97.1% (87.8%–99.5%) at the end of treatment (EOT) for the 200 and 800 mg groups, respectively (Supplementary Figure 2).

**Figure 1:**
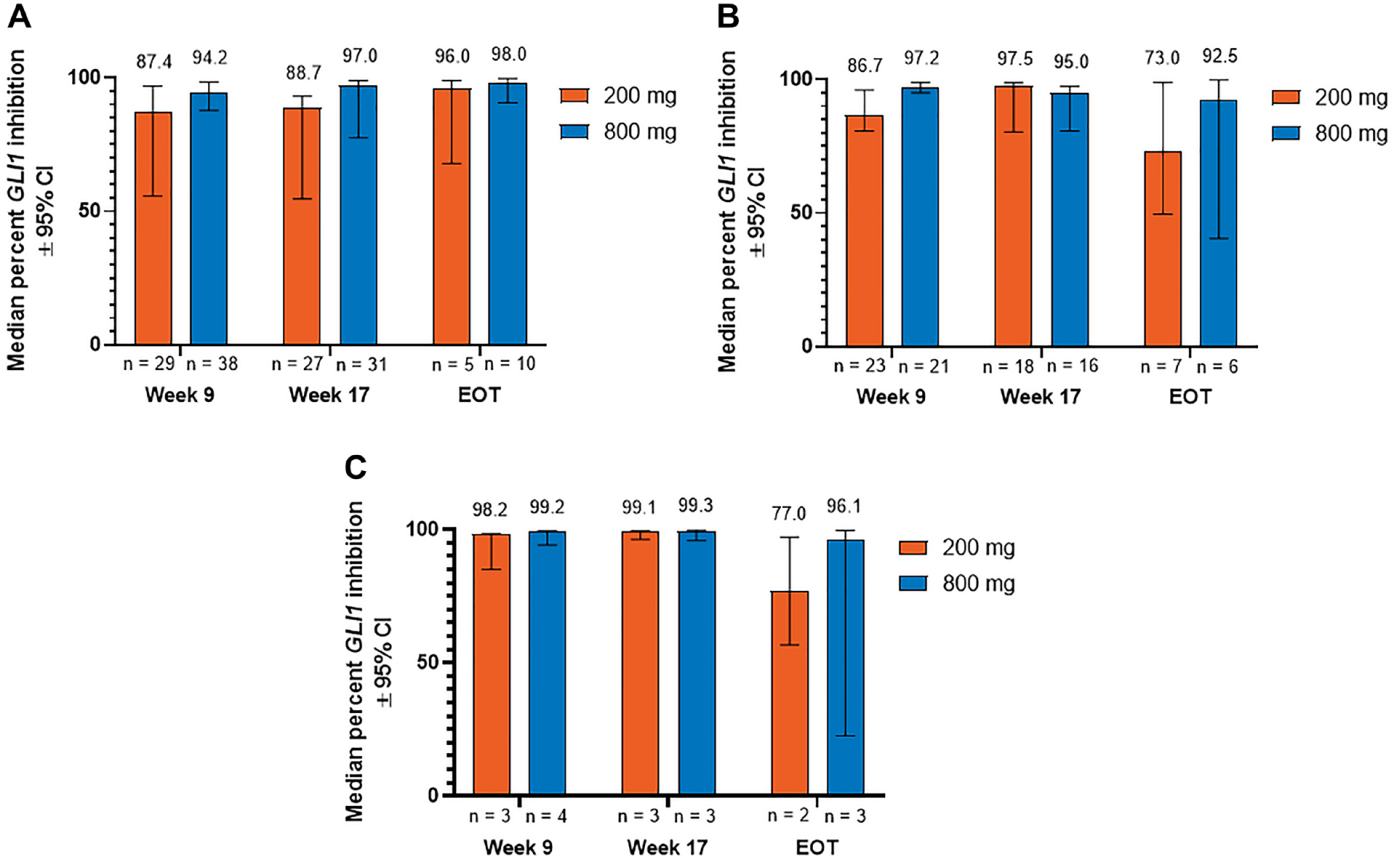
Percent inhibition of *GLI1* expression relative to baseline in patients with (**A**) aggressive laBCC, (**B**) nonaggressive laBCC, and (**C**) mBCC. BCC, basal cell carcinoma; CI, confidence interval; EOT, end of treatment; *GLI1*, *Glioma associated oncogene homolog 1*; laBCC, locally advanced BCC; mBCC, metastatic BCC.

When patients in the 2 dose groups were stratified by the type of BCC, median (95% CI) reduction in *GLI1* expression at week 9 was 87.4% (55.6%–96.9%) and 94.2% (87.8%–98.4%) for patients with aggressive laBCC, 86.7% (80.8%–96.1%) and 97.2% (95.0%–98.9%) for patients with nonaggressive laBCC, and 98.2% (85.1%–98.5%) and 99.2% (94.3%–99.6%) for patients with mBCC receiving sonidegib 200 and 800 mg QD, respectively (Figure 1). At week 17, median (95% CI) percent reduction was 88.7% (54.6%–93.0%) and 97.0% (77.5%–98.9%) for aggressive laBCC, 97.5% (80.3%–98.8%) and 95.0% (80.7%–97.5%) for nonaggressive laBCC, and 99.1% (96.4%–99.6%) and 99.3% (95.9%–99.9%) for mBCC in the 200 and 800 mg groups, respectively. Median (95% CI) reduction in *GLI1* expression at EOT was 96.0% (67.9%–98.9%) and 98.0% (90.6%–99.7%) for patients with aggressive laBCC, 73.0% (49.5%–98.9%) and 92.5% (40.5%–99.8%) for patients with nonaggressive laBCC, and 77.0% (56.8%–97.3%) and 96.1% (22.6%–99.7%) for patients with mBCC receiving sonidegib 200 and 800 mg QD, respectively.

### Subgroup analyses of GLI1 expression

**Figure 2:**
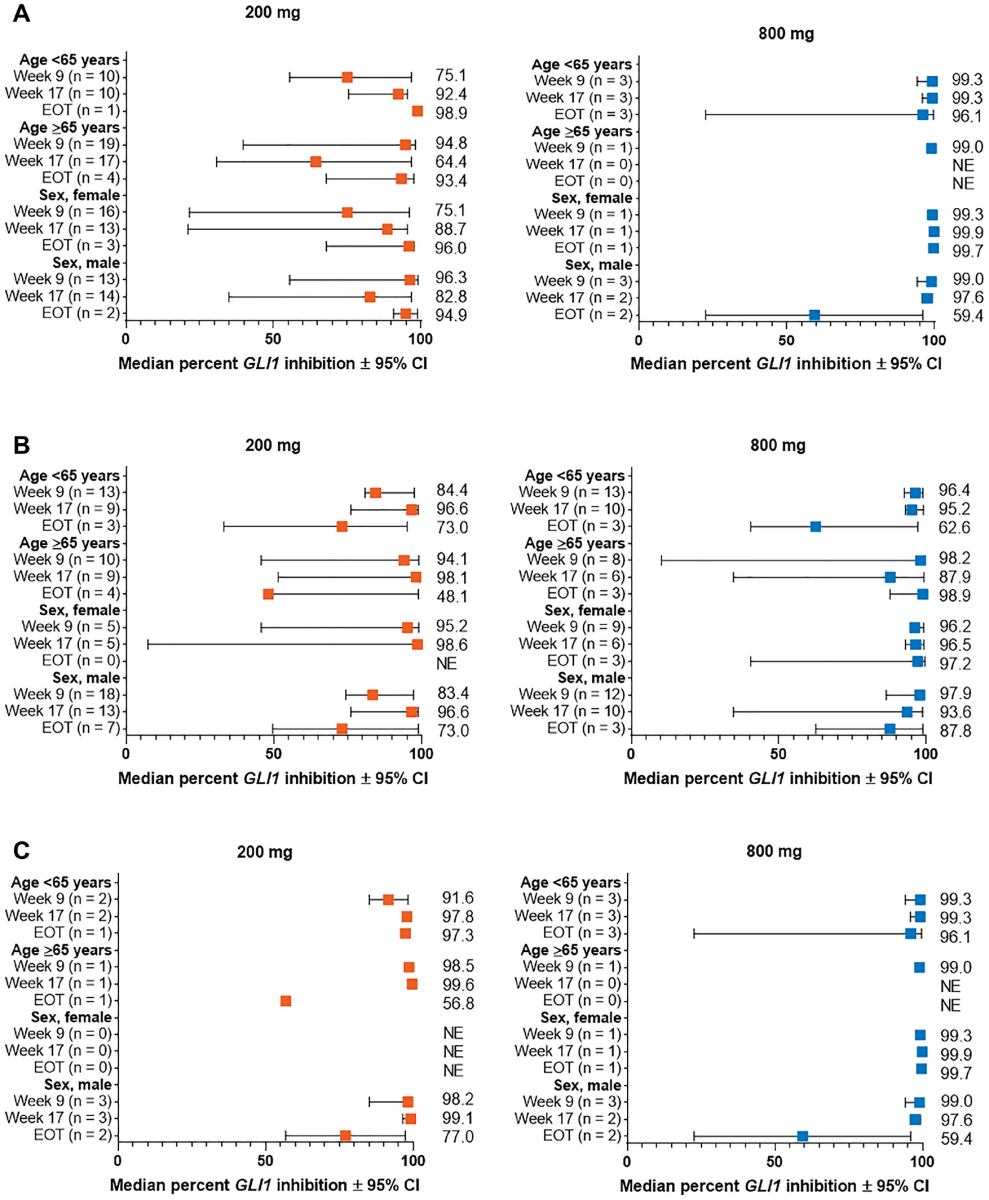
Percent change from baseline in *GLI1* expression in subgroups by demographic characteristics for patients with (**A**) aggressive laBCC, (**B**) nonaggressive laBCC, and (**C**) mBCC. BCC, basal cell carcinoma; CI, confidence interval; EOT, end of treatment; *GLI1*, *Glioma associated oncogene homolog 1*; laBCC, locally advanced BCC; mBCC, metastatic BCC; NE, not estimable.

**Figure 3:**
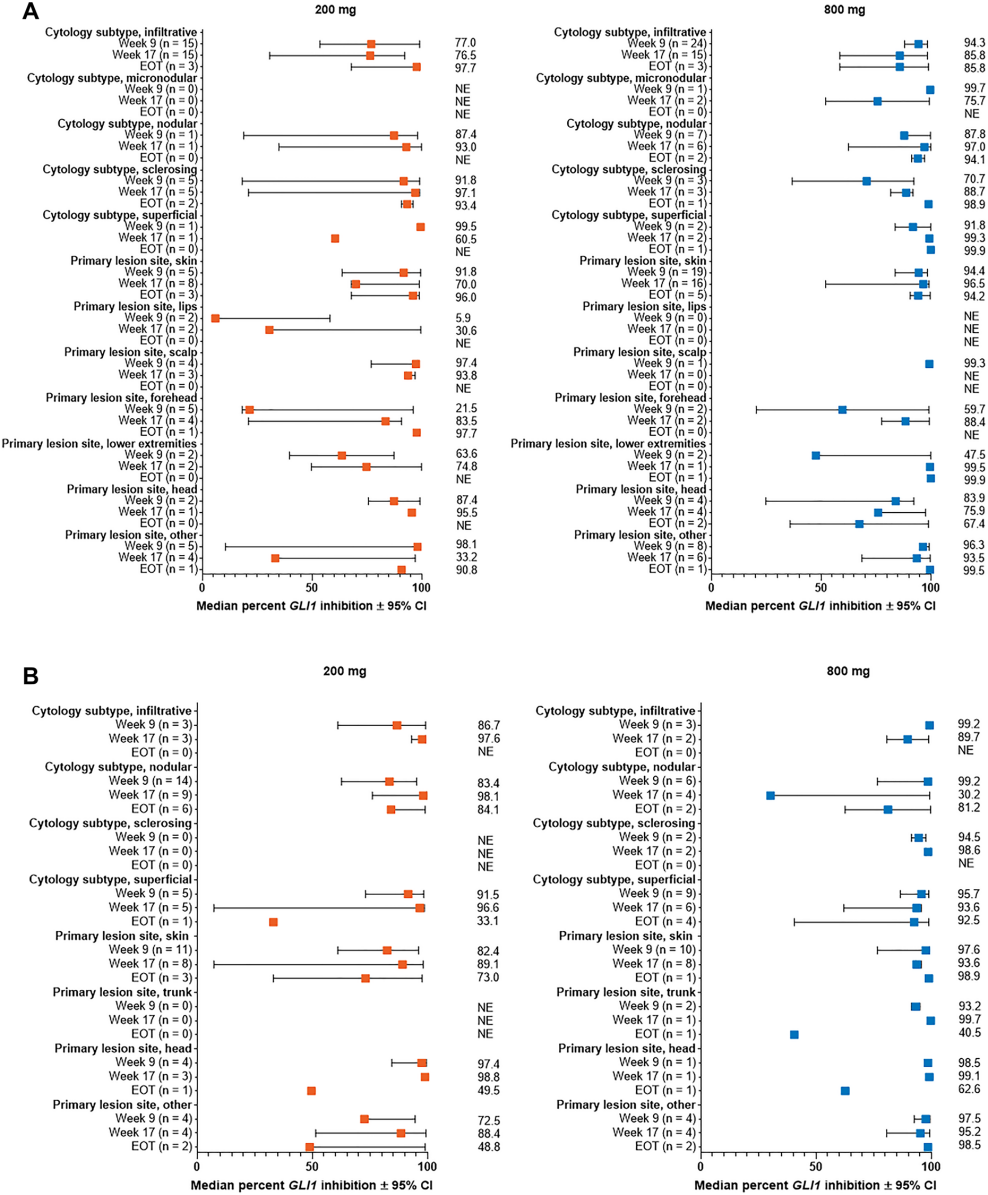
Percent change from baseline in *GLI1* expression in subgroups by lesion cytology and site for patients with (**A**) aggressive laBCC, (**B**) nonaggressive laBCC. Results are shown for subgroups with > 1 patient in either dose group. All subgroups for patients with mBCC had ≤ 1 patient. BCC, basal cell carcinoma; CI, confidence interval; EOT, end of treatment; *GLI1*, *Glioma associated oncogene homolog 1*; laBCC, locally advanced BCC; mBCC, metastatic BCC; NE, not estimable.

**Figure 4:**
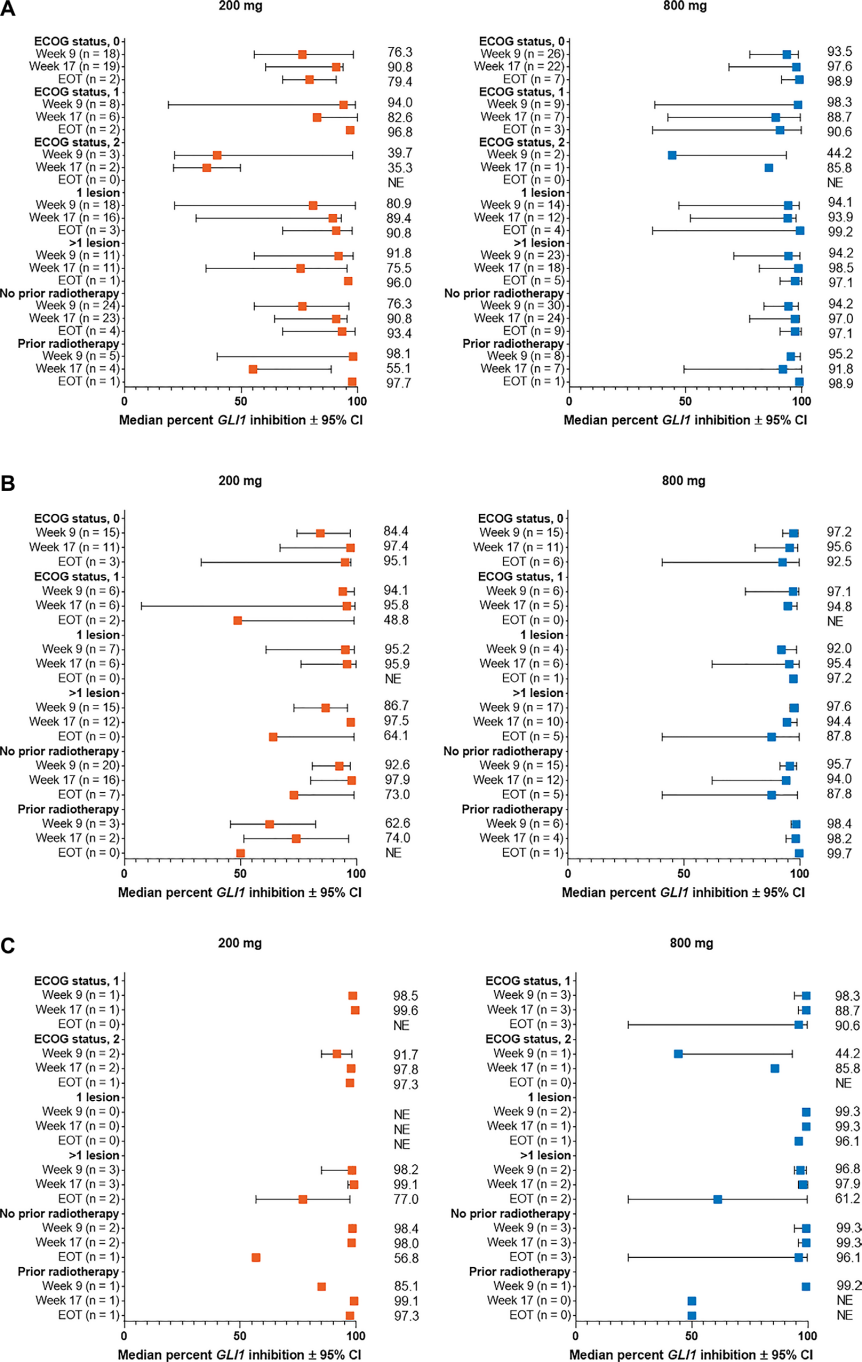
Percent change from baseline in *GLI1* expression in subgroups by Eastern Cooperative Oncology Group status, number of lesions, and prior radiotherapy for patients with (**A**) aggressive laBCC, (**B**) nonaggressive laBCC, and (**C**) mBCC. BCC, basal cell carcinoma; CI, confidence interval; EOT, end of treatment; *GLI1*, *Glioma associated oncogene homolog 1*; laBCC, locally advanced BCC; mBCC, metastatic BCC; NE, not estimable.

Marked reduction in *GLI1* expression from baseline was overall consistent between subgroups of patients with laBCC and mBCC stratified by demographic (Figure 2) and baseline clinical characteristics (Figures 3 and 4). Median percent reduction for patients with aggressive laBCC ranged longitudinally (week 9 to EOT; lowest and highest value shown) 75.1%–98.9% and 90.6%–97.5% for those < 65 years, and 64.4%–94.8% and 92.9%–98.9% for those ≥ 65 years receiving sonidegib 200 and 800 mg QD, respectively. For patients with nonaggressive laBCC, median percent reduction ranged longitudinally 73.0%–96.6% and 62.6%–96.4% in patients younger than 65 years, and 48.1%–98.1% and 87.9%–98.9% in patients ≥ 65 years receiving sonidegib 200 and 800 mg QD, respectively. Patients with mBCC achieved median percent reduction ranging longitudinally 91.7%–97.8% and 96.1%–99.3% for those younger than 65 years, and 56.8%–99.6% and 99.0% (data available for 1 patient for week 9 only) for patients ≥ 65 years receiving sonidegib 200 and 800 mg QD, respectively.

Patient subgroups stratified by BCC cytological subtype demonstrated relatively consistent reduction in *GLI1* expression from baseline across subtypes (Table 1).

**Table 1:** Percent inhibition of *GLI1* expression relative to baseline by cytological subtype

	200 mg QD			800 mg QD		
	laBCC		mBCC	laBCC		mBCC
	Aggressive	Nonaggressive		Aggressive	Nonaggressive	
**Basosquamous**	*n* = 0	*n* = 1	*n* = 2	*n* = 1	*n* = 0	*n* = 0
Week 9	—	99.1 (NE)	—	98.3 (NE)	—	—
Week 17	—	85.2 (NE)	—	99.3 (NE)	—	—
EOT	—	—	—	—	—	—
**Infiltrative**	*n* = 21	*n* = 3	*n* = 3	*n* = 28	*n* = 6	*n* = 3
Week 9	77.0 (53.7–99.1)	86.7 (61.0–99.1)	91.7 (85.1–98.2)	94.3 (88.0–98.4)	99.2 (98.0–99.5)	99.2 (99.0–99.3)
Week 17	76.5 (30.7–92.3)	97.6 (93.0–99.1)	97.8 (96.4–99.1)	85.8 (58.5–98.9)	89.7 (80.7–98.8)	99.9 (NE)
EOT	97.7 (67.9–98.9)	—	97.3 (NE)	96.9 (35.8–99.7)	—	99.7 (NE)
**Micronodular**	*n* = 0	*n* = 0	*n* = 0	*n* = 2	*n* = 0	*n* = 0
Week 9	—	—	—	99.7 (NE)	—	—
Week 17	—	—	—	75.7 (52.0–99.3)	—	—
EOT	—	—	—	—	—	—
**Multifocal**	*n* = 1	*n* = 0	*n* = 0	*n* = 0	*n* = 2	*n* = 0
Week 9	63.7 (NE)	—	—	—	99.1 (NE)	—
Week 17	75.5 (NE)	—	—	—	—	—
EOT	—	—	—	—	—	—
**Nodular**	*n* = 8	*n* = 14	*n* = 1	*n* = 9	*n* = 8	*n* = 1
Week 9	87.4 (18.8–98.2)	83.4 (62.6–95.2)	98.5 (NA)	87.8 (5.0–99.8)	98.5 (76.5–99.4)	94.2 (NE)
Week 17	93.0 (34.9–99.9)	98.1 (76.0–99.5)	99.6 (NA)	97.0 (62.4–99.9)	30.2 (93.2–99.3)	95.9 (NE)
EOT	—	84.1 (49.5–98.9)	—	94.1 (91.2–97.1)	81.2 (62.6–99.7)	22.6 (NE)
**Morpheaform**	*n* = 5	*n* = 0	*n* = 1	*n* = 3	*n* = 2	*n* = 0
Week 9	91.8 (18.2–99.1)	—	—	70.7 (36.7–92.2)	94.5 (91.4–97.6)	—
Week 17	97.1 (21.0–99.1)	—	—	88.7 (81.7–91.8)	98.6 (97.5–99.7)	—
EOT	93.4 (90.8–96.0)	—	56.8 (NE)	98.9 (NE)	—	—
**Superficial**	*n* = 1	*n **=*** 7	*n* = 1	*n* = 2	*n* = 11	*n* = 0
Week 9	99.5 (NE)	91.5 (73.0–98.3)	—	91.8 (83.7–99.9)	95.7 (86.5–98.9)	—
Week 17	60.5 (NE)	96.6 (7.3–98.6)	—	99.3 (99.2–99.5)	93.6 (62.1–95.6)	—
EOT	—	33.1 (NE)	—	99.9 (NE)	92.5 (40.5–98.9)	—
**Other**	*n* = 0	*n* = 0	*n* = 0	*n* = 1	*n* = 1	*n* = 1
Week 9	—	—	—	—	97.2 (NE)	99.6 (NE)
Week 17	—	—	—	—	95.6 (NE)	99.3 (NE)
EOT	—	—	—	—	—	96.1 (NE)

All data presented as median, % (95% CI), unless otherwise noted. CI, confidence interval; EOT, end of treatment; *GLI1*, *Glioma-associated oncogene homolog 1*; laBCC, locally aggressive basal cell carcinoma; mBCC, metastatic basal cell carcinoma; NE, not estimable due to 1 patient in group; QD, once daily.

### Association between GLI1 levels and efficacy outcomes

Substantial reductions in *GLI1* levels from baseline were observed in patients with disease control (complete response [CR], partial response [PR], or stable disease [StDis]), with median percent reduction ranging 74.5%–97.9% and 95.7%–98.0% at week 9, and 90.8%–99.5% and 96.1%–97.0% at week 17, for the 200 and 800 mg groups, respectively (Supplementary Table 2). However, a significant association was not observed between strength of *GLI1* repression and odds of tumor response (CR+PR), with hazard ratio (HR; 95% CI, *P*-value) for low vs high tumor inhibition of 1.4 (0.5–3.8, *P* = 0.4838) and 0.8 (0.3–2.0, *P* = 0.6627), for the 200 and 800 mg groups, respectively (Supplementary Table 3).

Overall, no significant association was observed between extent of *GLI1* inhibition and time to tumor response (TTR), with HR (95% CI, *P*-value) for low vs high expression of 0.9 (0.4–1.9, *P* = 0.4932) and 1.4 (0.7–2.8, *P* = 0.3148) for the 200 and 800 mg groups, respectively (Supplementary Table 4).

### Association between GLI1 levels and time to onset of grade ≥ 2 creatine kinase elevation

There was no overall significant association between extent of *GLI1* inhibition and time to onset of grade ≥ 2 creatine kinase (CK) elevation, with HR (95% CI, *P*-value) for low vs high expression of 0.6 (0.2–2.5, *P* = 0.3348) and 1.2 (0.5–2.6, *P* = 0.3348) for the 200 and 800 mg groups, respectively (Supplementary Table 5). Among patients with greater *GLI1* inhibition from baseline, those in the 800 mg dose group had an increased risk of grade ≥ 2 CK elevation, with HR (95% CI, *P*-value) vs the 200 mg dose of 2.3 (0.8–6.2, *P* = 0.0406).

## DISCUSSION

Results from this secondary analysis suggest that sonidegib treatment led to substantial reductions in *GLI1* levels from baseline across doses, BCC subtypes, examined time points, and demographic and baseline disease characteristics in patients with advanced BCC. Marked reductions in *GLI1* levels from baseline were observed in patients who achieved disease control with sonidegib, consistent with Hedgehog pathway inhibition.

The Hedgehog pathway plays a key role in regulating cell proliferation and differentiation during development and is involved in the maintenance and repair of many adult tissues including the skin, hair, muscles, and nervous system [19]. Upon activation of the pathway, a signaling ligand of the Hedgehog family binds the transmembrane receptor *PTCH1* [20]. This causes the G-protein coupled receptor *SMO*—sonidegib’s target in the pathway—to dissociate from *PTCH1* and migrate to the primary cilium, where it releases the cytoplasmic sequestration of the *GLI* family of transcription factors by *Suppressor of Fused* through a multistep signaling cascade [20]. Noncanonical pathways of *GLI1* activation have also been reported, including by the Ras and p53 families of tumor suppressors [21]. Aberrant *GLI1* activation promotes tumor growth, migration, and angiogenesis [21].

Despite the overall durable efficacy of sonidegib treatment observed in the BOLT study, a subset of patients develop resistance to HHIs that can be either primary or acquired after initial response to treatment [22]. Moreover, although *GLI1* suppression is dose- and exposure-dependent, a reduction of *GLI1* expression did not always correlate with tumor response in the phase 1 efficacy and safety study for sonidegib, most likely due to limited sample size, indicating resistance may develop despite *GLI1* inhibition [10]. The strong—but not complete—inhibition of *GLI1* expression in this biomarker analysis is consistent with the possibility that patients with low *GLI1* inhibition are resistant to sonidegib. However, there was no significant correlation between sonidegib efficacy and the strength of *GLI1* inhibition. This is possibly due to the study not being sufficiently powered to confirm this correlation. Additionally, inhibition of *GLI1* in the different patient subgroups examined was uniformly strong, and none of the examined demographic and baseline clinical characteristics showed strong correlation with sonidegib resistance.

Animal and cell-line cancer models studied the impact of sonidegib on Hedgehog pathway activity. In human primary glioblastoma initiating cells, sonidegib reduces levels of *GLI1*, *GLI2*, *PTCH1*, and *PTCH2* messenger ribonucleic acid (RNA) and protein, as well as *GLI1* and *GLI2* translocation into the nucleus [23]. Epistasis testing using sonidegib in combination with direct *GLI1* and *GLI2* inhibition by RNA interference revealed the effect of sonidegib on Hedgehog pathway downstream targets, including upregulation of the cell death mediators, Fas, Death receptor (DR)4, and DR5, and downregulation of the cell proliferation mediators B-cell lymphoma 2 and Platelet-derived growth factor receptor A [23]. In human renal cell carcinoma lines, sonidegib inhibited *GLI1* and *GLI2* and reduced cell proliferation in combination with everolimus or sunitinib, whereas direct inhibition of *GLI1* and *GLI2* in combination with everolimus or sunitinib had no impact on proliferation, suggesting that other targets downstream of *SMO* may play a role in tumor response [24].

This analysis found no significant association between the magnitude of *GLI1* inhibition and time to onset of grade ≥ 2 CK elevation—suggesting that CK elevation is not influenced by the extent of *GLI1* inhibition. CK elevation is a common treatment-emergent AE observed in patients treated with Hedgehog inhibitors. While the exact mechanism responsible for muscle spasms and increased CK levels in patients receiving HHIs is not completely understood, muscle spasms are considered to be linked with paradoxical activation of the *SMO*/calcium/AMP-activated protein kinase axis, and the inhibition of *SMO* signaling leads to an influx of calcium into muscle cells [25]. The pivotal clinical studies of vismodegib (Erivedge^®^, Genentech, South San Francisco, CA), an HHI indicated for the treatment of advanced BCC, did not include CK monitoring; however, muscle spasms were reported in 71.2% of patients [26]. All currently approved HHIs have the same mechanism of action and similar safety profiles; it is thought that CK elevation and muscle spasms are HHI class-effect AEs.

Limitations of the current study include the lack of statistical power to assess specific biomarker-related hypotheses. All biomarker analysis should be considered exploratory and hypothesis-generating, and all *P*-values were nominal or were adjusted for multiplicity only at the biomarker level within a specific model or analysis. *GLI1* was the only examined biomarker, and data on the expression of other prominent members of the pathway were not collected. Additionally, *GLI1* expression as related to reduction in tumor mass was not directly evaluated in this analysis. Subsequently, decreases in tumor size may have contributed to reductions in *GLI1* levels measured following treatment.

In summary, the reduction of *GLI1* expression is consistent with potent inhibition of the Hedgehog pathway by sonidegib in patients with laBCC and mBCC. These results support further clinical studies on the impact of sonidegib on Hedgehog pathway biomarkers.

## MATERIALS AND METHODS

### BOLT study design

This randomized, double-blind, adaptive phase 2 multicenter study adhered to the ethical principles of the Declaration of Helsinki and received approval for its protocol and all amendments from an Independent Ethics Committee or Institutional Review Board at each study site. Written informed consent was obtained from all patients before any study-specific procedures.

Study design is described in detail elsewhere and is briefly summarized here and in Supplementary Figure 2. The study enrolled men and women age ≥ 18 years with a histologically confirmed diagnosis of mBCC or laBCC (not amenable to radiation therapy, curative surgery, or other local therapies) and a World Health Organization (WHO) performance status ≤ 2. Patients were randomized 1:2 to receive sonidegib 200 or 800 mg QD until disease progression, unacceptable toxicity, withdrawal of consent, study termination, or death.

### BOLT efficacy assessments

Tumor response was evaluated with Response Evaluation Criteria in Solid Tumors (RECIST) version 1.1 for patients with mBCC. For laBCC, the standard RECIST 1.1 criteria are inadequate, since posttreatment morphological changes such as ulceration, cyst formation, and scarring may confound tumor response evaluation. A modified (m)RECIST protocol was developed to assess tumor response in laBCC, integrating central histological review, one-dimensional localized soft-tissue magnetic resonance imaging (MRI) along the lesion’s longest diameter per RECIST 1.1, and bidimensional color photography measurements per WHO guidelines.

The primary efficacy endpoint was ORR per central review. Secondary efficacy assessments included TTR and best overall response (either CR, PR, StDis, progressive disease, or unknown). Best overall response was assessed by central review according to mRECIST in patients with laBCC and RECIST 1.1 in patients with mBCC. mRECIST criteria includes a combination of MRI scans, color photography, and baseline and follow-up histopathology data for patients to assess tumor response.

### BOLT safety assessments

Safety assessments included AEs monitored throughout the study, coded using the medical Dictionary for Regulatory Activities version 19.0, and assessed for toxicity using the National Cancer Institute Common Terminology Criteria for Adverse Events version 4.03. CK levels were assessed prior to starting treatment or within 72 hours of the first dose, then weekly during the first 2 months, and every 4 weeks thereafter while on study treatment.

### Biomarker assessments

Biopsies were collected from accessible lesions at Screening, weeks 9 and 17 predose, and within 21 days after the last dose of study drug. For patients with multiple lesions, no specific lesion was designated for biopsy, and any lesion could be used. Presence of tumor tissue in biopsy samples was histologically confirmed prior to biomarker analysis.

*GLI1* expression at Screening was used as baseline measurement. Gene expression at all examined time points was assessed using reverse transcriptase polymerase chain reaction, in terms of the number of reaction cycles needed to reach the threshold amount of product. Expression of *GLI1* at each time point was normalized to the housekeeping gene ubiquitin C, and fold and percent change from baseline of normalized *GLI1* expression were computed.

Percent change from baseline in *GLI1* expression was stratified by type of BCC (aggressive and nonaggressive laBCC and mBCC), age, sex, number of lesions at baseline, cytology subtype, primary site of cancer, prior radiotherapy, and Eastern Cooperative Oncology Group performance status. Association between *GLI1* expression and select efficacy and safety assessments was evaluated, including best overall response, TTR, and time to onset of grade ≥ 2 CK elevation.

### Statistical analyses

The ITT population included all randomized patients. The subset of the ITT population with valid biomarker samples comprised the biomarker population used for all biomarker analyses. Data collected up to 6 months after the last patient randomization date were included in the analysis. All statistical evaluations were exploratory since the study was not powered to assess specific hypotheses regarding change in *GLI1* expression. Since inhibition of the Hedgehog pathway with sonidegib should result in *GLI1* inhibition, postbaseline records with > 100% change in *GLI1* levels from baseline were considered outliers and were excluded from analyses and models.

Visual assessments of strip plots determined the relationship between baseline *GLI1* levels or changes from baseline in *GLI1* levels and clinical efficacy outcomes. Percent change in *GLI1* levels was summarized using descriptive statistics (mean, 95% CI, median, 95% distribution-free CI, SD, quartiles, and range). Longitudinal analyses of *GLI1* expression were performed using a linear mixed model, including fold change in *GLI1* expression from baseline as a response variable and visit, treatment group, and visit-by-treatment interaction as covariates. Median percent change from baseline and 95% CI for each dose group and time point were computed based on model-based least squares mean fold change estimates.

Cox proportional hazards models determined associations between baseline *GLI1* levels or changes from baseline in *GLI1* levels, and TTR and time to onset of grade ≥ 2 CK elevation. Kaplan-Meier curves and log-rank test–based *P*-values for the difference in time-to-onset curves were produced for groups defined by dose level and categorized *GLI1* levels. Normalized baseline *GLI1* levels were categorized as low or high based on the median (both doses combined). Percent change in *GLI1* from baseline at week 9 or 17 was categorized as low (greater inhibition) or high (lesser inhibition) based on the third quartile (both doses combined).

## SUPPLEMENTARY MATERIALS



## Abbreviations

AE: adverse event
BCC: basal cell carcinoma
BOLT: Basal cell carcinoma Outcomes with LDE225 (sonidegib) Treatment
CI: confidence interval
CK: creatine kinase
CR: complete response
DR: death receptor
EOT: end of treatment
*GLI1*: Glioma-associated oncogene homolog 1
HHI: Hedgehog pathway inhibitor
HR: hazard ratio
ITT: intent-to-treat
laBCC: locally advanced BCC
mBCC: metastatic BCC
mRECIST: modified RECIST
MRI: magnetic resonance imaging
ORR: objective response rate
PR: partial response
*PTCH1*: Patched 1
QD: once daily
RECIST: Response Evaluation Criteria in Solid Tumors
RNA: ribonucleic acid
*SMO*: Smoothened
SD: standard deviation
StDis: stable disease
TTR: time to tumor response
WHO: World Health Organization
