# Supplementary material for: Expression of Glioma-associated oncogene homolog 1 as biomarker with sonidegib in advanced basal cell carcinoma
Source: Oncotarget. 2020 Sep 15;11(37):3473–83. doi: 10.18632/oncotarget.27735 (PMC7500103; doi:10.18632/oncotarget.27735)
Supplement: Supplementary file 1 [file oncotarget-11-3473-s001.pdf]

Expression of Glioma-associated oncogene homolog 1 as biomarker with sonidegib in advanced basal cell carcinoma

SUPPLEMENTARY MATERIALS

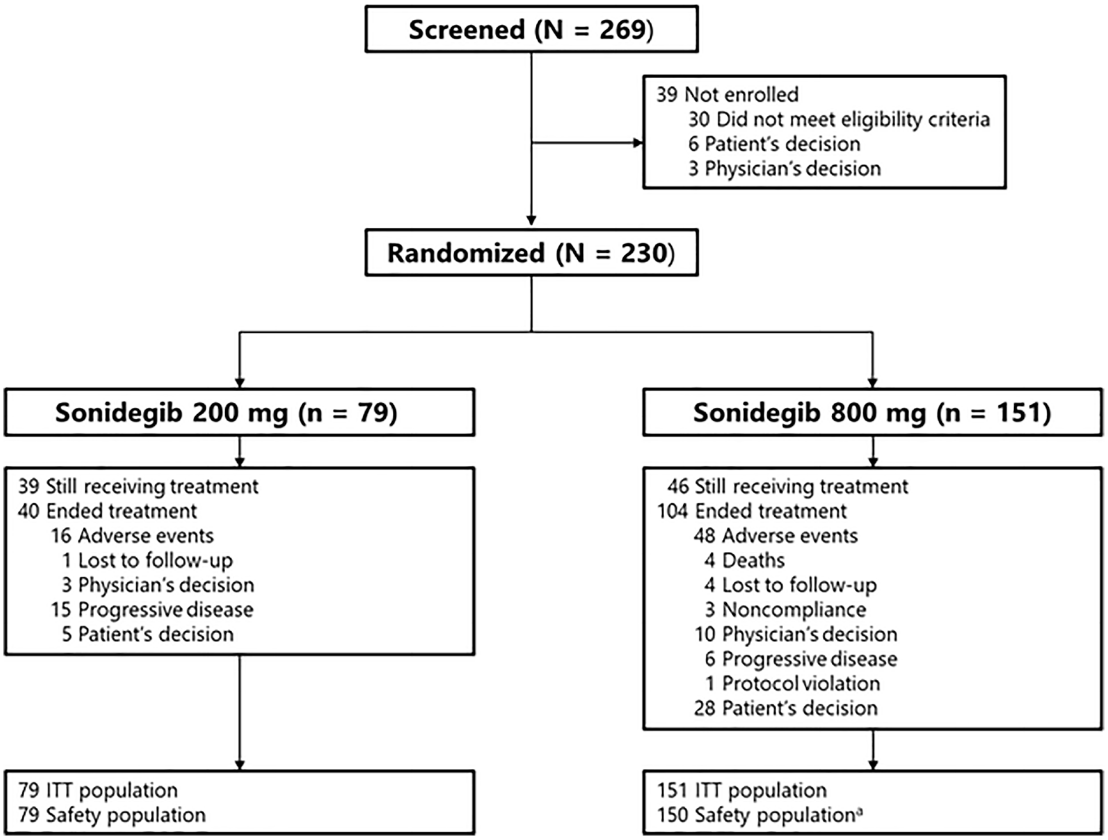

Supplementary Figure 1: Patient disposition at data cutoff for biomarker analysis (6 months). [a]One patient randomized to sonidegib 800 mg did not receive treatment. ITT, intent-to-treat.

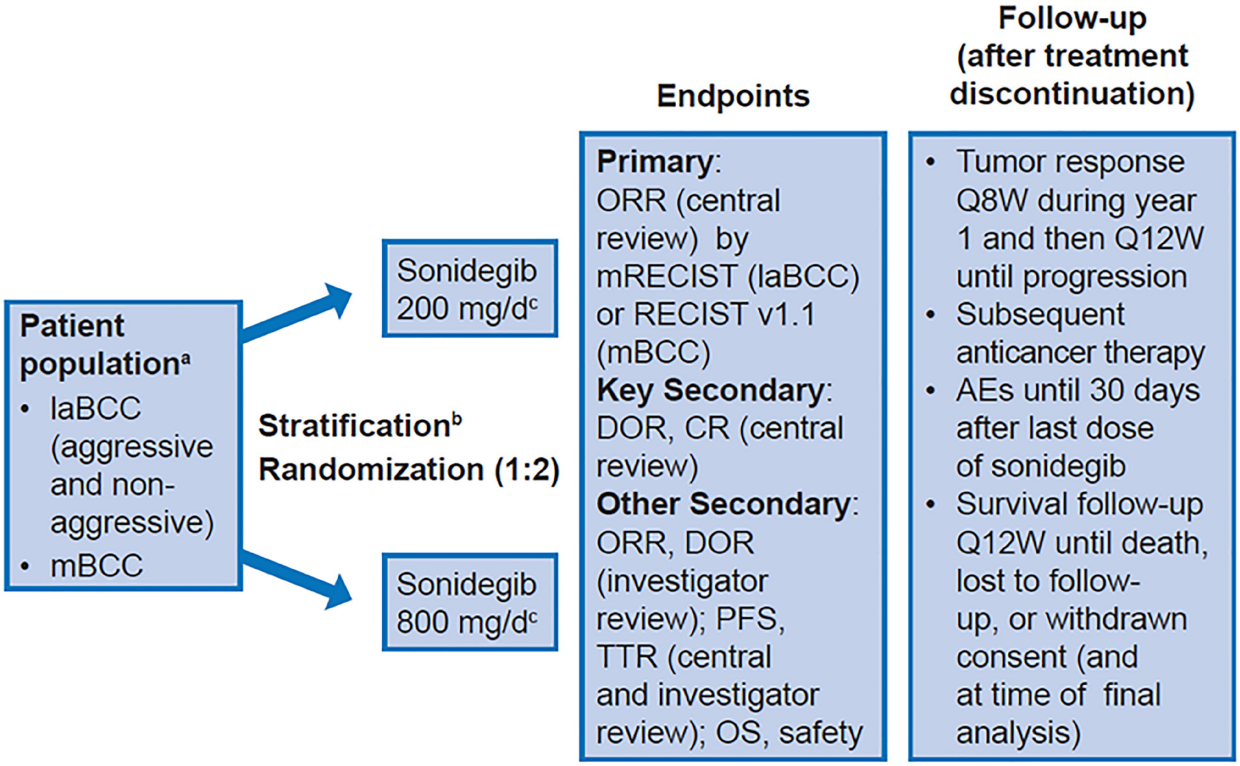

Supplementary Figure 2: BOLT study design. [a]Patients previously treated with sonidegib or other HHI were excluded. [b]Stratification was based on stage, disease histology for patients with laBCC (nonaggressive vs aggressive), and geographic region. [c]Treatment was continued until disease progression, unacceptable toxicity, death, study termination, or withdrawal of consent. AE, adverse event; BCC, basal cell carcinoma; BOLT, Basal Cell Carcinoma Outcomes with LDE225 (sonidegib) Treatment; CR, complete response; DOR, duration of response; HHI, Hedgehog inhibitor; laBCC, locally advanced BCC; mBCC, metastatic BCC; mRECIST; modified RECIST; ORR, objective response rate; OS, overall survival; PFS, progression-free survival; Q8W, every 8 weeks; Q12W, every 12 weeks; RECIST, Response Evaluation Criteria in Solid Tumors; TTR, time to tumor response.

Supplementary Table 1: Patient characteristics of the biomarker and intent-to-treat populations

	Biomarker population		ITT population	
	**200 mg (*n* = 67)**	**800 mg (*n* = 83)**	**200 mg (*n* = 79)**	**800 mg (*n* = 151)**
**Sex**, *n* (%)				
Female	25 (37.3)	29 (34.9)	31 (39.2)	55 (36.4)
Male	42 (62.7)	54 (65.1)	48 (60.8)	96 (63.6)
**Age,** years				
Mean (SD)	65.6 (15.6)	64.0 (14.9)	65.6 (15.7)	63.6 (14.6)
Range	25–92	24–93	25–92	24–93
**Stage of disease,** *n* (%)				
laBCC	61 (91.0)	76 (91.6)	66 (83.5)	128 (84.8)
mBCC	6 (9.0)	7 (8.4)	13 (16.5)	23 (15.2)
**Best overall response at 6 months**, *n* (%)				
CR	2 (3.0)	0	2 (2.5)	0
PR	27 (40.3)	34 (41.0)	31 (39.2)	49 (32.5)
StDis	32 (47.8)	32 (38.6)	39 (49.4)	68 (45.0)
PD	1 (1.5)	1 (1.2)	1 (1.3)	1 (0.7)
Unknown	5 (7.5)	16 (19.3)	6 (7.6)	31 (20.5)

BCC, basal cell carcinoma; CR, complete response; ITT, intent-to-treat; laBCC, locally advanced BCC; mBCC, metastatic BCC; PD, progressive disease; PR, partial response; SD, standard deviation; StDis, stable disease.

Supplementary Table 2: Percent reduction of *GLI1* inhibition by best overall response category

BOR category	Week 9		Week 17	
	**200 mg** *n* = 79	**800 mg** *n* = 151	**200 mg** *n* = 79	**800 mg** *n* = 151
**CR**				
n (%)	2 (2.5)	0	1 (1.3)	0
Median	97.9	NE	99.5	NE
(95% CI)	(97.5–98.3)	(NE)	(NE)	(NE)
**PR**				
n (%)	23 (29.1)	31 (20.5)	23 (29.1)	26 (17.2)
Median	74.5	95.7	90.8	97.0
(95% CI)	(58.3–93.7)	(88.0–99.0)	(64.4–95.5)	(85.8–98.8)
**StDis**				
n (%)	26 (32.9)	23 (15.2)	21 (26.6)	15 (9.9)
Median	93.2	98.0	96.6	96.1
(95% CI)	(80.9–98.2)	(94.2–98.5)	(76.5–98.1)	(62.1–99.3)
**PD**				
n (%)	1 (1.3)	0	1 (1.3)	0
Median	98.1	NE	10.2	NE
(95% CI)	(NE)	(NE)	(NE)	(NE)
**Unknown**				
n (%)	3 (3.8)	9 (6.0)	2 (2.5)	9 (6.0)
Median	95.2	92.6	94.2	91.8
(95% CI)	(82.4–96.2)	(34.5–99.7)	(88.7–99.8)	(34.7–97.5)

BOR, best overall response; CI, confidence interval; CR, complete response; *GLI1*, glioma-associated oncogene 1; NE, not estimated; PD, progressive disease; PR, partial response; StDis, stable disease.

Supplementary Table 3: Association between *GLI1* inhibition and best overall tumor response

Comparison	Odds ratio	95% CI	*P*-value
GLI1 low vs high, 200 mg group	1.4	(0.5–3.8)	0.4838
GLI1 low vs high, 800 mg group	0.8	(0.3–2.0)	0.6627
800 mg vs 200 mg, *GLI1* low	1.2	(0.5–3.1)	0.6987
800 mg vs 200 mg, *GLI1* high	0.7	(0.3–1.8)	0.4441

GLI1 low and high groups were defined based on whether baseline *GLI1* normalized expression was higher or lower than the median baseline normalized expression. The baseline median was computed using combined data from both dose groups. CI, confidence interval; *GLI1*, glioma-associated oncogene 1.

Supplementary Table 4: Association between *GLI1* inhibition and time to tumor response

Comparison	Odds ratio	95% CI	*P*-value
GLI1 low vs high, 200 mg group	0.9	(0.4–1.9)	0.4932
GLI1 low vs high, 800 mg group	1.4	(0.7–2.8)	0.3148
800 mg vs 200 mg, *GLI1* low	1.5	(0.7–3.2)	0.3574
800 mg vs 200 mg, *GLI1* high	1.0	(0.5–2.0)	0.4649

GLI1 low and high groups were defined based on whether baseline *GLI1* normalized expression was higher or lower than the median baseline normalized expression. The baseline median was computed using combined data from both dose groups. CI, confidence interval; *GLI1*, glioma-associated oncogene 1.

Supplementary Table 5: Association between *GLI1* inhibition and time to onset of grade ≥2 creatine kinase elevation

Comparison	Odds ratio	95% CI	*P*-value
GLI1 low vs high, 200 mg group	0.6	(0.2–2.5)	0.3348
GLI1 low vs high, 800 mg group	1.2	(0.5–2.6)	0.3348
800 mg vs 200 mg, *GLI1* low	4.2	(1.2–14.6)	0.0188
800 mg vs 200 mg, *GLI1* high	2.3	(0.8–6.2)	0.0406

GLI1 low and high groups were defined based on whether baseline *GLI1* normalized expression was higher or lower than the median baseline normalized expression. The baseline median was computed using combined data from both dose groups. CI, confidence interval; *GLI1*, glioma-associated oncogene 1.
